# Supplementary material for: Standardized metrics can reveal region-specific opportunities in community engagement to aid recruitment in HIV prevention trials
Source: PLoS One. 2020 Sep 17;15(9):e0239276. doi: 10.1371/journal.pone.0239276 (PMC7498108; doi:10.1371/journal.pone.0239276)
Supplement: Attachment — Submitted filename: Response to Reviewers_15July.docx [file pone.0239276.s001.docx]

July 1, 2020

Jose A. Bauermeister, MPH, PhD
Academic Editor
PLOS ONE

Manuscript Number: PONE-D-20-04045
Title: Standardized Metrics Can Reveal Region-Specific Opportunities in Community Engagement to Aid Recruitment in HIV Prevention Trials

To the editorial staff of PLOS ONE:

We wish to thank the assigned editors for their thorough review of our manuscript. We appreciate the thoughtful feedback and have incorporated many of the suggested revisions to improve the manuscript. We have addressed each of the reviewers’ concerns in a point by point fashion below, with their comments in bold print and our responses immediately following. We have provided line numbers for revisions where relevant.

**1. Please ensure that your manuscript meets PLOS ONE's style requirements, including those for file naming. The PLOS ONE style templates can be found at**

[**https://journals.plos.org/plosone/s/file?id=wjVg/PLOSOne_formatting_sample_main_body.pdf**](https://journals.plos.org/plosone/s/file?id=wjVg/PLOSOne_formatting_sample_main_body.pdf) **and**

[**https://journals.plos.org/plosone/s/file?id=ba62/PLOSOne_formatting_sample_title_authors_affiliations.pdf**](https://journals.plos.org/plosone/s/file?id=ba62/PLOSOne_formatting_sample_title_authors_affiliations.pdf)

Thank you for providing these links. We have revised our manuscript according to the format guidelines specified.

**2. Thank you for stating the following beneath the Acknowledgments Section of your manuscript:**

**“Funding
The HVTN 703/HPTN 081 and HVTN 704/HPTN 085 studies are supported by National Institute of Allergy and Infectious Diseases (NIAID) U.S. Public Health Service grants:
 UM1 AI068614 (Leadership and Operations Center: HIV Vaccine Trials Network),
 UM1 AI068618 (Laboratory 310 Center: HIV Vaccine Trials Network),
 UM1 AI068635 (Statistical and Data Management Center: HIV Vaccine Trials Network).
 UM1 AI068619 (Leadership and Operations Center: HIV Prevention Trials Network)
 UM1 AI068613 (Laboratory Center: HIV Prevention Trials Network)
 UM1 AI068617 (Statistical and Data Management Center: HIV Prevention Trials Network)
The content is solely the responsibility of the authors and does not necessarily represent the official views of the NIAID or NIH.”**

**We note that you have provided funding information that is not currently declared in your Funding Statement. However, funding information should not appear in the Acknowledgments section or other areas of your manuscript. We will only publish funding information present in the Funding Statement section of the online submission form.**

**Please remove any funding-related text from the manuscript and let us know how you would like to update your Funding Statement. Currently, your Funding Statement reads as follows:**

**“The authors received no specific funding for this work.”**

**Please clarify the sources of funding (financial or material support) for your study. List the grants or organizations that supported your study, including funding received from your institution.**

a. **State what role the funders took in the study. If the funders had no role in your study, please state: “The funders had no role in study design, data collection and analysis, decision to publish, or preparation of the manuscript.”**
b. **If any authors received a salary from any of your funders, please state which authors and which funders.**
c. **If you did not receive any funding for this study, please state: “The authors received no specific funding for this work.”**

**Please include your amended statements within your cover letter; we will change the online submission form on your behalf.**

Thank you for noting this inconsistency. We have revised our manuscript accordingly. To clarify, the funding that supported this work was part of the overall funding for the Leadership Operations Centers that our two networks receive from the National Institute of Allergy and Infectious Diseases. Our network leadership groups were supportive of the work, and our taking the time to collect the data and analyze it. Our salaries are part of the Leadership Operations Center awards for our NIAID-sponsored networks. The authors received no specific funding for this work because it was considered part of our networks’ core operations work.

**3. We noted in your submission details that a portion of your manuscript may have been presented or published elsewhere:**

**“Portions of this manuscript were presented as an oral abstract at the HIVR4P conference in Madrid, Spain in October, 2018. The abstract from the conference presentation was published in the conference program and made available on their website. The manuscript expands on this presentation substantively.”**

**Please clarify whether this conference proceeding was peer-reviewed and formally published. If this work was previously peer-reviewed and published, in the cover letter please provide the reason that this work does not constitute dual publication and should be included in the current manuscript.**

To clarify, the work presented at the 2018 HIVR4P conference consisted of a 10 minute oral presentation. We presented a brief summary of our methods, the raw data shown in Tables 1 and 2, brief conclusions about the value of region-specific approaches, and acknowledgements. All of the analysis, discussion, and recommendations for the future are new material that has never previously been presented. To our knowledge, the conference had panels of reviewers for submitted abstracts to determine which would be accepted for presentation, but this is not as rigorous as we believe the peer review by your journal constitutes. The accepted abstracts from the conference were published in an open-access online supplement in *AIDS Research and Human Retroviruses.* We have uploaded a copy of the accepted conference abstract and the oral presentation for your review on the manuscript submission portal.

**4. One of the noted authors is a group, 'HVTN 703/HPTN 081 and HVTN 704/HPTN 085 Protocol Teams'.**

**In addition to naming the author group, please list the individual authors and affiliations within this group in the acknowledgments section of your manuscript.**

**Please also indicate clearly a lead author for this group along with a contact email address.**

Thank you for this comment. The listing of the members of the protocol team as an author is a naming convention used by our networks, but we feel it is not practical to list the more than 50 members of these teams as authors. We have revised the list of authors accordingly, and have moved the protocol team members to the acknowledgements section of the manuscript.

The corresponding lead author will be Gail Broder, [gbroder@fredhutch.org](mailto:gbroder@fredhutch.org), as noted in lines 13-17.

**Reviewers' comments:**

**Reviewer #1: In the Introduction it reads like the study’s effectiveness at adhering to the principles of respect, mutual understanding, integrity, etc. will be evaluated in this study. It isn’t until the very end that it becomes evident that the purpose of this study is to assess the efficiency of the recruitment strategies. This should be made explicit earlier on and more clearly worked into the logic of the study rationale in the introduction.**

Thank you for this observation. We have reorganized the Introduction to move this information earlier in the manuscript, and have tried to provide more thorough descriptions and definitions of community engagement, and how recruitment is an element of Good Participatory Practice.

**It is not very evident that this paper is basically evaluating the effectiveness of different recruitment strategies in different settings. The focus on the GPP guidelines takes away from this. Authors should consider focusing on how these guidelines apply to recruitment practices and how recruitment using GPP guidelines differs from other types of recruitment. It is really not clear why GPP guidelines are important when the meat of this study is basically in the answer to the question, “how did you hear about this study?”.**

We thank the reviewers for this input. We have made revisions to the Introduction section that we hope more clearly explain why we feel that this community engagement work falls within the GPP guidelines, and how recruitment efforts are one element of GPP implementation. Lines 106-113 in particular speak to this.

**Reviewer #2**

**There are some potential discrepancies in characterizations of the approach and its implementation, and interpretations of the results. Finally, the manuscript would benefit significantly from delving into the rich field of research, including empirical research as well as the few descriptive studies cited (none in the discussion), to characterize the many tensions and challenges that underlie what is presented as a rather neutral and uncomplicated process of community engagement (CE). In addition, there are many studies of recruitment practices. Engaging with the existing literature would make the findings and insights gained much more valuable and serve to better advance the field.**

We agree with the reviewers that this is a rich area for research that would benefit from additional study. In our review of the literature around recruitment we found that few articles tie recruitment to the GPP principles nor their implementation. Thus, our premise that there are no standardized metrics for evaluating the implementation of GPP, either in its entirety or in the small part that we have focused on, is still appropriate. However, we did expand on this in our Discussion section (lines 277-286) to better characterize the history of meaningful community engagement.

**There are far too many acronyms, particularly for a general scientific audience that reads PLOS One: suggest using some but limiting substantially.**

Thank you for this suggestion. We have reduced the use of acronyms substantially.

**It would be helpful to include and discuss relevant literature on community engagement in, particularly, HIV prevention trials, much of which is omitted. At least these need to be referenced and addressed in the discussion.**

Thank you for this suggestion. We have edited the manuscript to provide a brief overview of the benefits of community engagement broadly, and in engaging communities in recruitment specifically (lines 138-156). Please also refer to lines 277-286 where our expanded literature search is reflected.

**Line 72: what is a “bnAb”?**

We note that bnAb was defined at first use in line 67, but in keeping with the suggestion above to reduce acronym use, we have removed all uses of this acronym and spelled out broadly neutralizing antibody fully when the term is used. **Line 77: break down n=2701 into n’s for cisgender and transgender men, and transgender women. (Also, one wonders if there are differentially effective CE and recruitment methods for different subpopulations?)**

We agree with the reviewers that information broken down by sub-populations would be important information to have, however, it was not part of the data that was collected in this initial effort and thus we cannot provide the requested breakdown. We have noted this as a study limitation and recommended it as an area for future study (lines 347-355).  **Line 86: broader CE than? Usual HVTN/HPTN practices? Or?**

Thank you for catching this error. This should have said “broad community engagement.” The text has been corrected.
 **Line 89: this is surely a substantial list of principles. However, it is unclear how each of these were addressed in your analysis. I do not recall mention of or disaggregation of these principles in the results or discussion.**

Thank you for this comment. Upon further reflection, we have deleted the sentence in lines 88-89 that addressed the specific elements of GPP because it was tangential to the study we conducted. We have also rearranged the placement of text that was previously included as lines 164 - 174. Please see the relocated text in lines 100-113 that we believe address the reviewers’ concern. **Also, what principles were omitted? And why?**

As stated above, getting into this depth regarding the GPP guidelines is not central to the work we did. We have removed this text. **Lines 90-96: While the GPP are certainly widely discussed guidelines, they are not without challenges; and a number of articles have evoked or discussed these. This needs to be acknowledged here, or in the discussion section. See list at end.**

We agree with the reviewers that there is much to be said about the GPP guidelines and their associated challenges. We included reference to this in lines 96-99.
 **Line 99: Too many acronyms. Are the CRSs considered local? Or beyond? What about NCEP staff? The question arises as to how many local staff are permanent; and how many international staff are permanent.**

We have removed many of the acronyms throughout the manuscript. The group of authors of this manuscript comprise the Network Community Engagement Program staff, and we are all permanent, full time employees. Some of us are based in Johannesburg, South Africa, while others are in the US. Lines 114-116 note that we are permanent staff. The clinical research sites are indeed what we consider to be local, as they work directly with the communities where recruitment activities occur. We have added text to lines 147-148 to better characterize this. **Line 100: why does the CWG only include one CAB rep and one community educator? Some studies have identified challenges in local community staff raising concerns or speaking frankly with international staff and investigators, more so as the latter pay the salaries of local staff. See references below. Arguably this may not be such a fraught issue in discussing study recruitment; however this should be acknowledged in the discussion.**

Thank you for this comment. We have added lines 121-137 to better describe the Community Working Groups and their composition. As the reviewers have noted, lines 135-137 note that study leaders are not part of the group, helping to create an environment where people can speak freely and without fear of repercussions.

**There seems to be a discrepancy between the statement on 119 (“underlying assumption that community members have the best insight regarding which recruitment strategies work best in their local cultural and geographic context”, and line 131, “NCEP staff for HVTN and HPTN developed a list of recruitment strategies based on their experiences working with the sites” and shared the lists with CWG members who “revised them for accuracy.” This may reflect a need to better describe the process; but why did the initial lists not emerge from “community members” if they are the experts? Might having lists pre-arranged by HVTN and HPTN staff influence the listing and choices of community members?**

Thank you for noting this discrepancy. We realized that text from an older draft had been included in the submitted manuscript, rather than the corrected text we had intended to use. This section has been corrected to accurately reflect the genesis of the list of recruitment strategies (lines 187-193).

**Lines 135-139: Here is an interesting example of different approaches taken by African CWG and Americas and Swiss CWG to characterize referral sources. Since you are advocating “common metrics”, how might these differences influence the metric devised? Should there be flexibility underlying the metrics and their application to enable different sites/site groups to apply them more accurately? How far does the flexibility go?**

We agree with the reviewers that flexibility is critical to successful recruitment. The Network Community Engagement Program staff always advocate for sites to adapt strategies to be appropriate for their local environment, which continues to ground the work in Good Participatory Practice and consulting with local stakeholders. Thus, comparisons between sites are informative, but do not necessarily suggest that one site has used a strategy better than another site. This is why we have presented the data in aggregate by region, rather than listing site data individually. In our Discussion section we have noted that our data are a starting point for the development of metrics, and one of our conclusions is that each research site must build on their knowledge of the local community to tailor strategies for their environment through the monitoring of their own local site data. In our work with local sites, we have found that flexibility is not mutually exclusive from the development of metrics that can meaningfully evaluate implementation of common standards.  **Lines 152-157: It would be informative to disaggregate numbers “found ineligible to enroll” and those who were “eligible but decided not to participate”? Those numbers are treated differently in clinical trials; and they could have different ramifications for recruitment practices: i.e., targeting the ‘wrong’ population vs. challenges in willingness to participate.**

While we agree with the reviewers that this would be interesting data, it was not part of our dataset and thus is not something that we have available for analysis. We anticipate that more will be learned about this once the studies are unblinded and the full analysis is presented, as reasons for “screen failure” were captured in a case report form for both studies.  **Later you do mention the need to include cost effectiveness analysis in future studies, which is fine. However, you might specifically mention that raw numbers of screening to enrollment ratios per recruitment strategy might not necessarily support an evidence informed decision to prioritize one over the other. For ex., if “transit advertising” were relatively much less expensive than face-to-face outreach, arguably the former might be an acceptable strategy, or some combination thereof.**

Thank you for this observation. We agree with the reviewers and have elaborated on our conclusion regarding an analysis of cost effectiveness (lines 376-382). **The complete lack of references and discussion of the present results in the context of many other studies of CE in HIV prevention trials and global health more broadly, as well as studies of recruitment, is a shortcoming and a lost opportunity to engage these findings with the existing literature. For ex., what are the challenges of “consulting with the community to identify recruitment strategies most appropriate for each region”? Who represents “the community”? Who decides on the representatives? From whom do the community representatives and community staff draw salaries? Are they permanent or temporary staff? What happens if different representatives do not agree? Arguably pastors and gay and transgender people do not likely agree on many important issues, which may impact on willingness of the latter to speak openly; and the recruitment strategies and target communities identified. See references below.**

As noted in our responses above, we did do an expanded literature search and have revised the manuscript accordingly. We agree with the reviewers, but the questions noted in their comments are part of a wider research scope than we sought to address in our study. In the HVTN and HPTN, the issues raised by the reviewers are part of the community engagement work undertaken by each local Community Advisory Board in consultation with the local site staff, who are employed by the clinical research sites. Work to address these issues is addressed in each site’s Annual Plan for Community Engagement. The members of the AMP Study Community Working Groups are representatives drawn from these local site staff and local Community Advisory Boards, thus they are a representative body that could provide appropriate input into the development of our project. We expanded on this in lines 114-156. **While this study does present helpful ideas and data on using community processes to identify metrics to identify preferred and effective recruitment strategies, what are some of the challenges in harmonizing metrics amidst cultural, geographical, and economic differences? In the context of stigma, criminalization of PLHIV and at-risk communities in some sites? Vast differences in wealth and power between trial sponsor nations and LMIC in which many trials are conducted? This is not to lessen the importance or potential utility of the findings; but it would make them more interesting and applicable in the real world if some of the most relevant literature and challenges were acknowledged, with discussion of how the development of such metrics might address or adapt to challenges in the face of disparate cultures, geographies, wealth and power that underlie the realities of global health research and HIV prevention and other clinical trials.**

We agree with the reviewers that these are important considerations that merit further exploration. We have revised our Discussion section to characterize this as a limitation of our study, and have included a recommendation that this be the subject of future research (lines 347-363). **See below: CE References, many of which are highly relevant:**

We thank the reviewers for providing this very helpful list of additional references, which we have reviewed. There were many helpful ideas among these papers, and we have revised our Discussion section accordingly (lines 277-286).

**-de Wet, A, Swartz, L, Kagee, A, et al. (2019): The trouble with difference: Challenging and reproducing inequality in a biomedical HIV research community engagement process, Global Public Health, DOI: 10.1080/17441692.2019.1639209

-Kagee, A., De Wet, A., Kafaar, Z. et ak, (2020). Caveats and pitfalls associated with researching community engagement in the context of HIV vaccine trials. Journal of Health Psychology, 25(1), 82–91.** [**https://doi.org/10.1177/1359105317745367**](https://doi.org/10.1177/1359105317745367) **-Lavery, James V. et al. Towards a framework for community engagement in global health research. Trends in Parasitology, Volume 26, Issue 6, 279 – 283. DOI:** [**https://doi.org/10.1016/j.pt.2010.02.009**](https://doi.org/10.1016/j.pt.2010.02.009) **-Molyneux, S., Sariola, S., Allman, D., et al. (2016). Public/community engagement in health research with men who have sex with men in sub-Saharan Africa: Challenges and opportunities. Health Research Policy and Systems, 14, 65.

-Montgomery, C. M., Sariola, S., Kingori, P., & Engel, N. (2017). Global health and science and technology studies: Complacency and critique. Science and Technology Studies, 30(4), 1–13

-Newman PA, Rubincam C, Slack C,et al. (2015) Towards a Science of Community Stakeholder Engagement in Biomedical HIV Prevention Trials: An Embedded Four-Country Case Study. PLoS ONE 10(8): e0135937. doi:10.1371/journal.pone.0135937

-Newman, PA & Rubincam, C. (2014) Advancing community stakeholder engagement in biomedical HIV prevention trials: principles, practices and evidence, Expert Review of Vaccines, 13:12, 1553-1562, DOI: 10.1586/14760584.2014.953484

-Rubincam, C., et al. (2015): Taking culture seriously in biomedical HIV prevention trials: a meta-synthesis of qualitative studies, Expert Review of Vaccines, DOI: 10.1586/14760584.2016.1118349

-Thabethe, S., Slack, C., (2018). “Why Don’t You Go Into Suburbs? Why Are You Targeting Us?”: Trust and Mistrust in HIV Vaccine Trials in South Africa. Journal of Empirical Research on Human Research Ethics, 13(5), 525–536.** [**https://doi.org/10.1177/1556264618804740**](https://doi.org/10.1177/1556264618804740) **Recruitment (among many others)

-Horvath KJ, Nygaard K, Danilenko GP, et al. Strategies to retain participants in a long-term HIV prevention randomized controlled trial: lessons from the MINTS-II study. AIDS Behav. 2012;16(2):469‐479. doi:10.1007/s10461-011-9957-3

-Newman, PA, Duan, N, Roberts, K, et al. HIV Vaccine Trial Participation Among Ethnic Minority Communities: Barriers, Motivators, and Implications for Recruitment, JAIDS Journal of Acquired Immune Deficiency Syndromes: February 1st, 2006 - Volume 41 - Issue 2 - p 210-217 doi: 10.1097/01.qai.0000179454.93443.60

-Screening, recruiting and predicting retention of participants in the NIMH Multisite HIV Prevention Trial, AIDS, Supplement: February 1997 - Volume 11 - Issue - p S13-S19**

Thank you for the opportunity to revise our manuscript based on this helpful feedback.

With best regards,

Gail B. Broder, Corresponding Author
